# Subcutaneous administration, higher age and lower renal function are associated with erythrocyte methotrexate accumulation in Crohn’s disease: a cross-sectional study

**DOI:** 10.1186/s12876-022-02439-y

**Published:** 2022-07-30

**Authors:** M. M. van de Meeberg, M. L. Seinen, H. H. Fidder, M. Lin, B. Oldenburg, N. K. de Boer, G. Bouma, R. de Jonge, M. Bulatović Ćalasan, the Dutch Initiative on Crohn and Colitis (ICC)

**Affiliations:** 1 Department of Gastroenterology and Hepatology, University Medical Centre Utrecht, Utrecht, The Netherlands; 2 Department of Gastroenterology and Hepatology, Amsterdam UMC, Vrije Universiteit Amsterdam, AGEM Research Institute, Amsterdam, The Netherlands; 3 Department of Gastroenterology, OLVG, Amsterdam, The Netherlands; 4 Department of Clinical Chemistry, Amsterdam UMC, Amsterdam, The Netherlands; 5 Department of Rheumatology and Clinical Immunology, University Medical Centre Utrecht, Utrecht, The Netherlands

**Keywords:** Crohn’s disease, Methotrexate, Pharmacokinetics, Therapeutic drug monitoring

## Abstract

**Background:**

Methotrexate is an immunomodulatory drug for patients with Crohn’s disease. Erythrocyte MTX-polyglutamates (MTX-PG_1-5_) may be used for therapeutic drug monitoring (TDM) as MTX-PG is thought to mediate MTX’s efficacy. Information on determinants of the concentration of MTX-PG in patients with Crohn’s disease is lacking. We aim to identify clinical and biochemical determinants of the erythrocyte MTX-PG_1-5_ and MTX-PG_total_ concentration in patients with Crohn’s disease.

**Methods:**

Adults with Crohn’s disease on methotrexate treatment who visited the outpatient clinic of Amsterdam UMC were included. Erythrocyte MTX-PGs were measured by tandem mass spectrometry.

**Results:**

Nineteen patients were included, with a median duration of MTX use of 77 months (range 7–202). Twelve patients received MTX monotherapy, whereas 7 patients were on concomitant TNF-α inhibitors. The mean dose of MTX was 15.5 mg (SD ± 2.8) and 12 (63%) patients used subcutaneous MTX. MTX-PG_1-5_ were successfully measured in 18 patients, showing substantial variability in concentrations of MTX-PG_total_ and individual species. The median MTX-PG_total_ was 117.1 nmol/L (range 46.4–258.7) with preferential accumulation of MTX-PG_3_ (43.1 nmol/L, range 15.3–96.1). Patients on subcutaneous compared to oral MTX had higher median MTX-PG_(4,5)_ levels (55 versus 9 nmol/L,* p* = 0.01). Higher age (β = 0.71) and lower estimated glomerular filtration rate (β = − 0.52) were associated with a significantly higher MTX-PG_total_ concentration (R^2^ = 0.60, *p* = 0.001).

**Conclusion:**

MTX-PG concentrations display a considerable inter-individual variability. Higher MTX-PG accumulation is associated with subcutaneous administration, higher age, and lower renal function in Crohn’s disease patients.

**Supplementary Information:**

The online version contains supplementary material available at 10.1186/s12876-022-02439-y.

## Background

Methotrexate (MTX) is an effective drug for both induction [1] and maintenance of remission in patients with Crohn’s disease (CD) [2, 3]. MTX is also prescribed in combination with tumour necrosis factor (TNF)-α- inhibitors to suppress anti-drug antibody formation [4]. Despite its relatively favourable safety profile, clinical effectiveness, and cost-effectiveness [5, 6], 37% and 26% of patients discontinue MTX monotherapy in the first year because of lack of response or side effects, respectively [7]. This is reflected by the observation in cohort studies that MTX is prescribed in only 3.3% of Dutch CD patients and in 1.5 to 5.9% of elderly American CD patients [8, 9].

Contrary to many other drugs used in the treatment of CD, MTX administration is based on a standard dosing regimen. Efficacy and avoidance of side effects may be further optimized by an individualized treatment. A prerequisite for such optimization is determination of the biologically active MTX metabolites through therapeutic drug monitoring (TDM). Currently, however, there is no established method for TDM of MTX, which can be explained by its relatively complex metabolism.

Low-dose MTX is eliminated from plasma in 24 h as MTX-PG_1_ (with one glutamate group attached) and is incorporated intracellularly by transporters such as the reduced folate carrier (RFC). Intracellularly, additional glutamate groups (MTX-PG_2-7_) are added by folylpolyglutamate synthetase (FPGS). In a competing reaction, the MTX-PGs are deconjugated by ɣ-glutamyl hydrolase (GGH), returning MTX to its monoglutamate form, which is pumped out of the cell by the ATP-binding cassette (ABC) family of transporters [10]. The balance between import, export, glutamylation and deconjugation determines the MTX-PG chain length within the cell. A higher degree of polyglutamylation results in cellular retention of MTX, as longer MTX-PGs cannot be released from the cells by ABC drug efflux transporters, and thereby, potentially, lead to an increased efficacy through stronger inhibition of target enzymes. The most important target enzymes of MTX-PGs, dihydrofolate reductase (DHFR), thymidylate synthase (TS), and 5-aminoimidazole-4-carboxamide ribonucleoside (AICAR) transformylase, inhibit purine and pyrimidine synthesis through folate antagonism and/or upregulation of anti-inflammatory adenosine [11]. Considering the abovementioned mechanism of action, intracellular erythrocyte MTX-PG is a plausible TDM tool. Erythrocytes are easy to acquire and have a fixed MTX-PG concentration, which is a reflection of MTX-PG formation and accumulation in immature erythroblasts in the bone marrow. This is the case because proliferating and differentiating erythroblasts harbor FPGS activity leading to formation of MTX-PGs, whereas mature erythrocytes lack intrinsic FPGS activity, resulting in a fixed MTX-PG concentration (inherited from erythroblasts) [12]. Prior to implementing TDM of MTX in the treatment of CD patients, several prerequisites should be met. These include establishing interpatient variability of MTX-PG concentrations, identifying pharmacokinetic parameters, and establishing a relationship between MTX-PG concentration and efficacy.

In rheumatoid arthritis (RA) and juvenile idiopathic arthritis (JIA), we measured MTX-PGs in erythrocytes using a robust tandem mass spectrometry technique and showed that higher concentrations of MTX-PG were associated with lower disease activity [13–15]. Age, MTX dose, erythrocyte folate status, and FPGS rs4451422 wild-type genotype were pharmacokinetic parameters of the MTX-PG concentration in RA patients [16]. However, in studies with inflammatory bowel disease (IBD) patients, where erythrocyte MTX-PGs were quantified, pharmacokinetic parameters were not analysed thoroughly [17–21].

Therefore, our primary aim was to identify pharmacokinetic parameters of MTX-PG concentration with a special focus on the contribution of the route of administration. We expected subcutaneous MTX administration to be an important determinant since the bioavailability after oral administration averages 73% of that of subcutaneous administration in CD patients [22].

In this pharmacokinetic cross-sectional study, we measured MTX-PG concentrations in CD patients using tandem mass spectrometry to evaluate interpatient variability and identify determinants of the MTX-PG concentration.

## Methods

### Study design and patients

This was a single-centre cross-sectional study performed in an academic medical centre. All patients with CD over 18 years of age, receiving weekly MTX and visiting the outpatient clinic of the Department of Gastroenterology and Hepatology of Amsterdam UMC (The Netherlands) from May 2019 until February 2020, were eligible and asked to participate. The included patients underwent regular visits at the outpatient ward and were treated according to the accepted clinical practice, following national and local guidelines, determined by the physician.

### Laboratory measurements

Left-over routine EDTA whole-blood for hemocytometric and digital imaging analysis was used for this study. The sampling time (the moment between blood withdrawal and MTX intake/injection) was not standardized since the inclusion of patients took place during regular outpatients ward visits. It is also very likely that in this study the sampling time point did not influence the MTX-PG concentrations as median duration of MTX use was long (77 months, Table 1) and MTX-PGs reached their steady-state within 6 months.^14^ On top of that, we measured MTX-PG concentrations in mature erythrocytes, which lack FPGS and GGH activity themselves [12]. EDTA whole blood was centrifuged (1700 × *g*) within 24 h and 500 µl erythrocyte pellets were stored at -80 °C. MTX-PGs were analysed from the cell pellet aliquots with an ultra-high-performance liquid chromatography-electrospray ionisation-tandem mass spectrometry-based assay (UPLC-ESI–MS/MS) using stable-isotope-labelled internal standards, as described previously [13]. In short, in each run, a 10-point calibration curve between 0 and 250 nM for each individual MTX-PG was included. The lower limit of quantitation was 1 nM (CV% < 20% and S/N > 10:1) for each MTX-PG and the assays were linear between 0 and 1000 nM.^12^ Concentrations of MTX-PGs were reported in nmol/L packed erythrocytes. MTX-PG_total_ is the sum of the five quantified subspecies of MTX-PG (MTX-PG_n*,*_). Erythrocyte and serum total folate were routinely measured using electrochemiluminescence immunoassay (Modular E170; Roche, Almere, The Netherlands) as described before [23]. The reported erythrocyte folate (in nmol/L) was corrected for plasma folate and estimated haematocrit.

**Table 1:** Patient characteristics (n = 19)

General characteristics	
Age*, years*	55 (21–71)
Female, n (%)	16 (84)
BMI, *kg/m*^*2*^	25 (21–37)
Current smoker, n (%)	5 (26)
Creatinin, *µmol/L*	74 (52–98)
eGFR, *ml/min/1.73 m*^*2*^	86 (66–152)
Haemoglobin, *mmol/L*	8.2 (6.4–9.5)
Erythrocyte folate, *nmol/L*	1630 (728–2283)

Characteristics of Crohn’s disease	
Age at diagnosis, n (%) ≤ 16 years 17–40 years > 40 years	4 (21) 7 (37) 8 (42)
Location of disease, n (%) ileum colon ileum and colon	2 (11) 4 (21) 13 (68)
Disease behaviour, n (%) inflammatory structuring penetrating	11 (58) 5 (26) 3 (16)
Peri-anal disease, n (%)	6 (32)
Duration of disease, *years*	15 (2–54)

Disease activity	
Flare following PGA, n (%)	4 (21)
CRP, *mg/L*	2.2 (0.9–20)

Medication	
Subcutaneous use, n (%)	12 (63)
Duration of MTX use, *months*	77 (7–202)
Co-therapy with TNF-α inhibitor, n (%)	7 (37)
MTX dose, *mg/week,* n (%) 15 10; 20; 25	16 (84) 1 (5); 1 (5); 1 (5)
Folic acid dose, *mg/week,* n (%) 15 10 5 1.5	11 (58) 1 (5) 6 (32) 1 (5)

Data presented as median (minimum—maximum) unless stated otherwise

Units are *italicised*

### Data collection

Demographic, clinical, biochemical and endoscopic data were retrieved from the medical patient files using an electronic predetermined structured datasheet incorporated into Castor Electronic Data Capture (EDC) (version 2019.1, Amsterdam, The Netherlands). One author (MvdM) extracted all data and another author (MS) checked the extraction forms on accuracy.

Collected data included the following dichotomous determinants: sex, current smoking behaviour, TNF-α inhibitor co-administration and route of administration of MTX. BMI (Body Mass Index), age at inclusion, duration of MTX use, MTX dose, dose of folic acid supplementation, creatinine (µmol/L), and eGFR (estimated glomerular filtration rate by CKD-EPI, in ml/min/1.73 m^2^) were collected as continuous determinants. Furthermore, duration of disease, disease activity (assessed by the treating physician following the Physician Global Assessment, PGA: remission or flare), CRP (C-Reactive Protein, in mg/L), Hb (haemoglobin, in mmol/L), Montreal classification, co-medication for CD other than TNF-α inhibitors (thiopurines, ustekinumab, vedolizumab, mesalazine, corticosteroids, rectal therapy) and previous CD treatments were extracted from the patient files.

### Statistical analysis

Data were presented as numbers with percentages, medians with minimum and maximum (range) or means with standard deviations (SD), when appropriate. Variability of MTX-PG concentrations was presented as the coefficient of variation (CV, SD divided by mean multiplied by 100%). For non-normally distributed data, nonparametric tests including the Mann‐Whitney *U* test, Kruskal‐Wallis, and Fisher Exact test were used to test for differences between groups.

Correlations between the MTX-PGn concentrations were analysed with the Spearman’s rank correlation coefficient or Point-Biserial correlation coefficient when appropriate. Before identifying determinants of the MTX-PG_n_ concentration using univariable linear regression analysis, assumptions of normality, linearity and constant variance on MTX-PG_total_ concentrations were checked and were met. In regression analysis, MTX-PG_n_ concentration was used as target variable and determinant(s) as predictor variable. All beta’s (β) were standardised. Significant determinants at univariable analysis and at the comparison between two groups (for dichotomous determinants only) were used for analysis of the R^2^ and multivariable analysis. Multivariable analysis using stepwise selection was performed for only the MTX-PG_(4,5)_ and MTX-PG_total_ concentration. Based on the event per variable rule of 10 cases per determinant (with a range of 5 up to 20), we choose to select only two determinants and an interaction effect (if significant). We selected the model with the maximized adjusted R^2^.

All the analyses were two-tailed, and *P* values less than 0.05 were considered significant. Statistical analyses were performed in R version 4.0.3 [24].

## Results

### Patient characteristics

Nineteen out of 25 eligible outpatient clinic patients gave a written informed consent and were enrolled in the study. Four patients stopped MTX before inclusion, one patient did not give consent and one patient did not have a blood withdrawal at our hospital during the inclusion period. **Table ****1** summarises the main characteristics of the study population. Most (84%) patients were female. Almost half of the patients had previously used biologics (47%) and a part underwent intestinal surgery (37%). All patients had been using MTX for at least seven months. Twelve patients were using MTX monotherapy (63%), while in seven patients MTX was prescribed in combination with a TNF-α inhibitor (4 patients adalimumab, 3 patients infliximab). IBD co-medication prescribed, other than TNF- α inhibitors, were prednisolone (n = 1, < 10 mg/day orally) and rectal corticosteroids and/or 5-ASA applications (n = 2). Only four patients had active disease according to PGA.

### MTX-PG concentrations

**Fig. 1:**
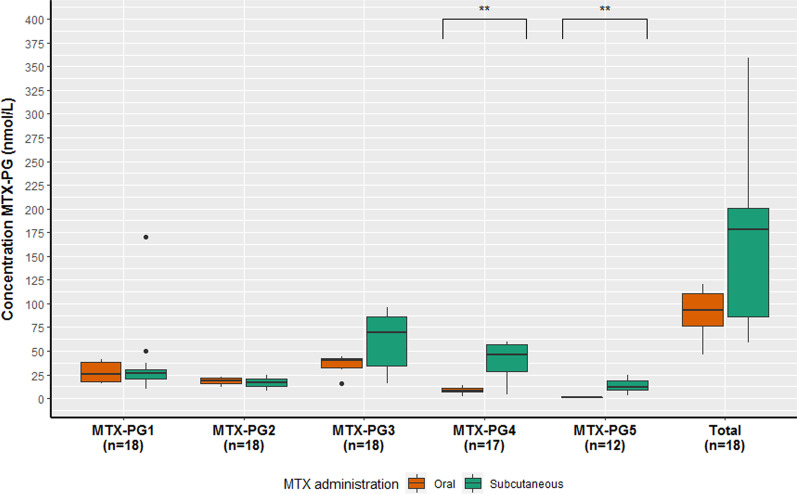
Boxplot of erythrocyte MTX-PG_n_ concentrations in nmol/L for CD patients using oral (orange) versus subcutaneous (green) MTX. Median MTX-PG concentrations (range) for all patients were as follows: MTX-PG_1_ 26.7 (9.9–170.1), MTX-PG_2_ 17.4 (7.5–24.8), MTX-PG_3_ 43.1 (15.3–96.1), MTX-PG_4_ 19.7 (2.4–60.1), MTX-PG_5_ 9.4 (1.1–24.1), MTX-PG_total_ 117.1 (46.4–258.7) nmol/L

We measured MTX-PG concentrations in 18 out of 19 patients (Fig. 1). We assume that one patient, with non-detectable MTX-PG_n_ concentrations, was not taking her medication and excluded her from further analysis. MTX-PG_4_ was measurable in 17 patients and MTX-PG_5_ in 12 patients.

The inter-individual variation (CV) are shown in Table 2. Remarkably, this variation was lower for patients on oral MTX than those on subcutaneous MTX (30.9 versus 50.0% for MTX-PG_total_).

**Table 2:** Coefficient of variation (%) of erythrocyte MTX-PG concentrations

	All (n = 18)	Oral MTX (n = 7)	Subcutaneous MTX (n = 12)
MTX-PG_1_	103.5	42.3	114.0
MTX-PG_2_	27.2	23.2	29.8
MTX-PG_3_	49.8	31.0	44.0
MTX-PG_4_	77.7	45.2	50.7
MTX-PG_5_	77.0	17.3	50.9
MTX-PG_total_	55.1	30.9	50.0

### Determinants of MTX-PG

The results of correlation and regression analysis for all variables with MTX-PG_n_ and MTX-PG_total_ concentration are displayed in Additional file 1: Table.

#### Dichotomous variables

The median MTX-PG_total_ concentration was 177.8 (58.8–358.7) for subcutaneous users versus 93.2 (46.4–120.8) nmol/L for oral users (*p* = 0.07; Fig. 1). Subcutaneous administration of MTX leads to a significantly higher MTX-PG_4_ and MTX-PG_5_ concentration compared to oral MTX administration (both p = 0.01). The median MTX-PG_(4,5)_ concentration of subcutaneous users was 55 (3.7–84.3) compared to 8.9 (2.4–15.0) nmol/L for oral users (*p* = 0.01). Significance for MTX-PG_4_ and MTX-PG_5_ remained even when only patients using MTX in a dose of 15 mg/week were included (n = 15). In univariable linear regression analysis, the β regression coefficient was significant for MTX-PG_3_ (β = 0.51, *p* = 0.03), MTX-PG_4_ (β = 0.69, *p* = 0.00), MTX-PG_5_ (β = 0.69, *p* = 0.01) and MTX-PG_total_ (β = 0.47, *p* = 0.049). Route of administration explained 48, 47 and 22% of the variability (R^2^) of the MTX-PG_4_, MTX-PG_5_, and MTX-PG_total_ concentration, respectively.

Comparison of males and females showed no significant difference in MTX-PG_n_ and MTX-PG_total_ concentration between the two groups (*p* = 0.65). However, this variable had just a significant correlation and β of 0.47 (*p* = 0.049) for MTX-PG_1_ concentration.

There was a trend towards higher MTX-PG_n_ concentrations in non-smokers (Additional file 2: Figure), which was significant for MTX-PG_2_ (median of 12.8 versus 18.4 nmol/L, *p* = 0.03), with a β of -0.54 (p = 0.02). The smoking status explained 29% of the variability of MTX-PG_2_.

No association of TNF-α inhibitor co-administration and MTX-PG_n_ concentration was found (*p* > 0.05).

#### Continuous variables

**Fig. 2:**
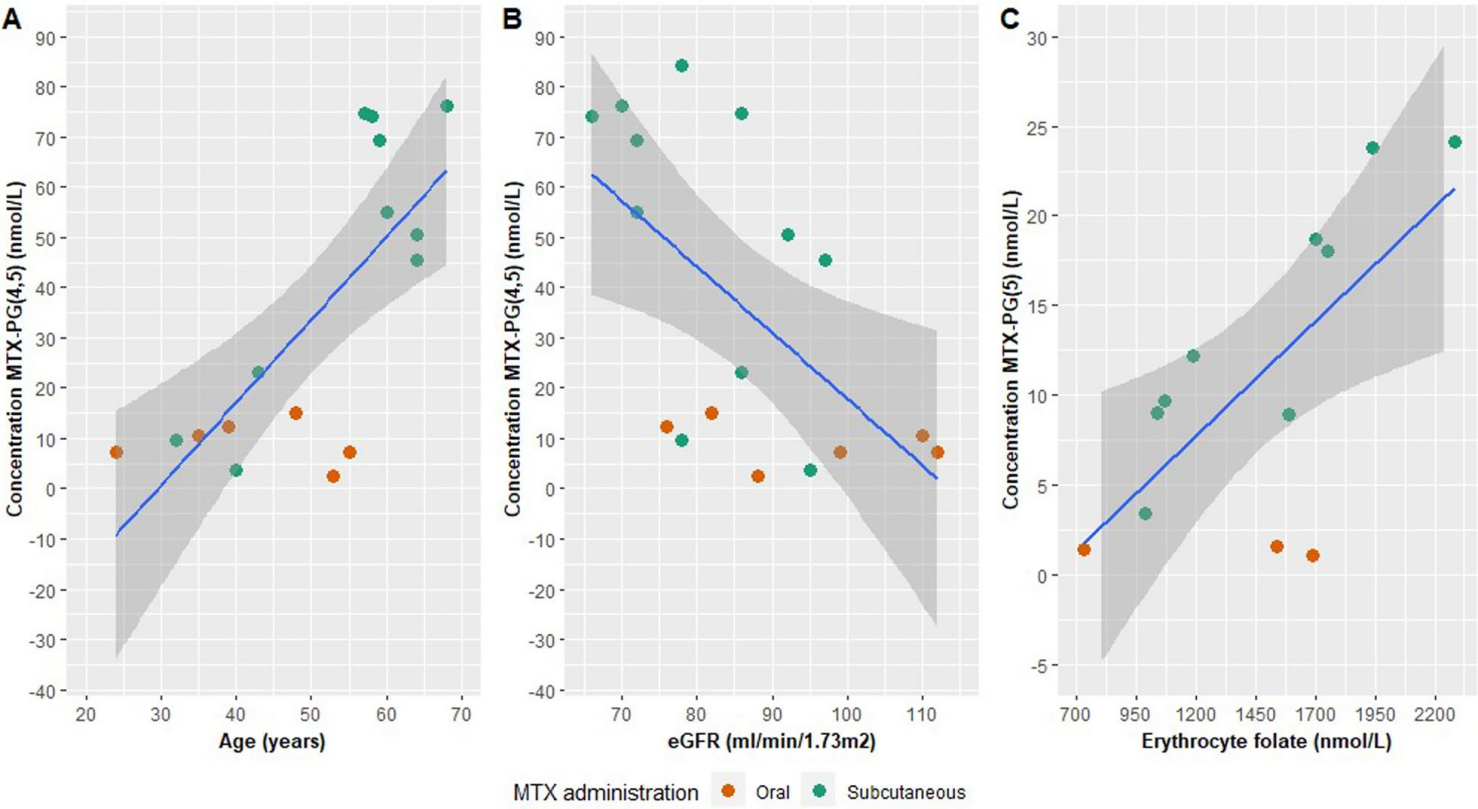
Linear regression of [A] MTX-PG_(4,5)_ concentration versus age [B] MTX-PG_(4,5)_ concentration versus eGFR [C] MTX-PG_5_ concentration versus erythrocyte folate concentration. β_[A]_ = 0.78 (*p* = 0.0002), β_[B]_ = − 0.58 (*p* = 0.01), β_[C]_ = 0.68 (*p* = 0.01). Results of individuals patients are displayed as a scatter plot, with orange dots for oral MTX users and green dots for subcutaneous MTX users

Older age was correlated with a higher MTX-PG_3_, MTX-PG_4_, MTX-PG_5_, and MTX-PG_total_ concentration (ρ_total_ = 0.80, *p* = 0.0001; β_total_ = 0.71, *p* = 0.001). This explained 50% of the variability of the MTX-PG_total_ concentration. Figure 2a shows the regression analysis of age and the MTX-PG_(4,5)_ concentration. Remarkably, subcutaneous MTX was prescribed more frequently in older patients. The median age of oral users was 39.0 (21–55) compared to 58.5 (32–71) years of subcutaneous users (*p* = 0.01).

A lower eGFR was correlated with a higher MTX-PG_4_ and MTX-PG_total_ concentration (ρ_total_ = − 0.60, *p* = 0.01; β_total_ = − 0.52, *p* = 0.03), which explained 37 and 27% of their variability. The regression analysis of eGFR and the MTX-PG_(4,5)_ concentration is shown in Fig. 2b. Age and eGFR were not significantly correlated with each other (ρ = − 0.43, *p* = 0.08), although there was a visible negative trend with respect to eGFR and age. Oral MTX users had a significantly better renal function (ρ = -0.54, *p* = 0.02).

There was a significant correlation and regression between the concentration of erythrocyte folate and MTX-PG_5_ (ρ = 0.66, *p* = 0.02; β = 0.68, *p* = 0.01), Fig. 2c. Erythrocyte folate explained 47% of the variability of MTX-PG_5_. Route of administration, age and eGFR were not significantly correlated with the erythrocyte folate concentration (*p* = 0.97, *p* = 0.34, *p* = 0.41, respectively).

BMI, duration of MTX use, MTX dose, folic acid supplement dose and creatinine did not correlate with MTX-PG_n_ concentrations. However, there was a trend toward higher MTX-PG_total_ concentrations in patients receiving a higher dose of MTX (Additional file 3: Figure).

#### Multivariable regression analysis

The MTX-PG_total_ concentration was best explained by the combination of age and eGFR, with an adjusted R^2^ of 0.48 (*p* = 0.003). Multivariable regression analysis did not provide a better explanation for the MTX-PG_total_ concentration variability than the determinant age alone in univariable analysis (R^2^ = 0.50, *p* = 0.001). There was a significant interaction effect between age and eGFR in the multivariable analysis. When corrected for this effect, the adjusted R^2^ improved to 0.60 (*p* = 0.001). Addition or replacement with the route of administration did not improve the significance and/or R^2^.

The MTX-PG_(4,5)_ concentration was best explained by the combination of the route of administration and age (R^2^ = 0.69, *p* < 0.001). There was a significant interaction effect between age (higher age) and the route of administration (subcutaneous users). When corrected for this effect, the adjusted R^2^ improved to 0.83 (*p* < 0.001). Addition of or replacement with eGFR or erythrocyte folate concentration to this analysis did not improve the significance and/or the R^2^.

## Discussion

In this pharmacokinetic study, we quantified erythrocyte MTX-PG_n_ concentrations in patients with CD and demonstrated large interpatient variability for all MTX-PG species using the robust UPLC-ESI–MS/MS technique with stable-isotope-labelled internal standards. We demonstrated that subcutaneous MTX use leads to a higher MTX-PG_(4,5)_ concentration. Age and eGFR were the most important determinants of the MTX-PG_total_ concentration explaining 60% of interpatient variability. We identified non-adherence in one patient.

**Fig. 3:**
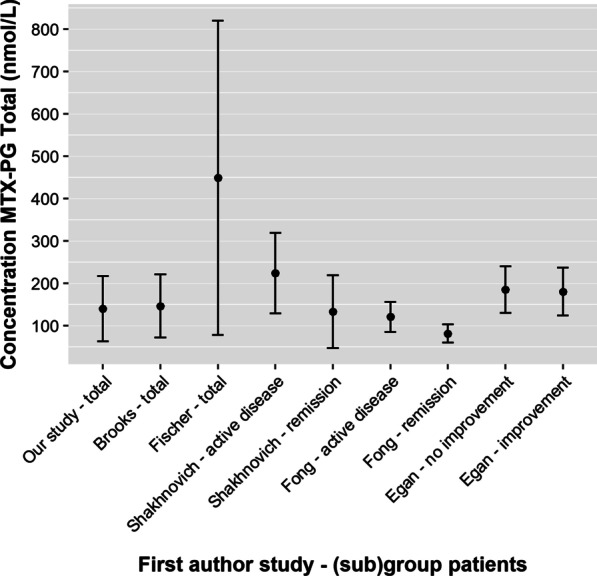
Mean erythrocyte MTX-PG_total_ concentration with standard deviation of our study population and other published IBD populations. MTX-PG concentrations expressed in nmol/8 × 10^12^ red blood cells as nmol/L, because the articles used packed or washed erythrocytes with a Red Blood Cell International System of Units Reference Range of 8 × 10^12^ red blood cells per litre.^17–19^

Thus far, five studies have measured erythrocyte MTX-PG concentrations in IBD patients (Table 3) [17, 19–21, 25, 26]. All studies had comparable, small, sample sizes (n = 12–30). Both patients with CD and ulcerative colitis were enrolled, except in the study by Fischer et al*.*[18] The mean MTX-PG_total_ concentration (140 ± SD 77 nmol/L) and inter-patient variability (CVs 27.2 to 103.5%) in our study was comparable with the data from most other studies, except for the study by Fischer et al*.* (Fig. 3). In line with other IBD (and RA) [15] studies, MTX-PG_3_ was our most abundant intracellular species (36.8% of MTX-PG_total_ compared to 32.5 and 43.0%) [19, 20].

**Table 3:** Overview of all available studies on erythrocyte MTX-PG concentrations and pharmacokinetics in patients with IBD

Article	Population					Methotrexate			Method		Pharmacokinetic results
First author, year, *journal*	N	From	Age (y)†	CD (%)	Active disease (%)	Oral (%)	Dose (mg/wk)	Co-therapy (%)	Study dsesign	Method of MTX-PG measurement	
Morrow, 2021, *Pharmaceuticals*^17^ & Shakhnovich, 2019, [A] *Gastroenterology*^25^	21	USA	5–21	90	48	86	5–25†	IFX (100)	Cross-sectional	HPLC–MS/MS of MTX-PG_1-6_	Relationship of dose and MTX-PG_total_ (ρ = 0.56) and MTX-PG_(3–5)_ (ρ = 0.51) No relationship of IFX concentration and MTX-PG_total_ or MTX-PG_3-5_ No relationship of route of administration and MTX-PG concentration^§^
Fischer, 2017, *Clinical Pharmacology and Drug Development*^18^ & Shivi, 2014, [A] *Inflammatory bowel disease*^26^	12	USA	30–68	100	42	17	25; at least 12 weeks	Steroid (25)	Cross-sectional	HPLC–MS/MS of MTX-PG_1-5_	Interpatient variation of 28-fold for MTX-PG_total_
Fong, 2014, [A] *Journal of Crohn’s and Colitis*^19^	21	UK	22–59	76	57	90	17 ± 1^ǂ^	IFX (57) ADA (14)	Retrospective cohort	HPLC of MTX-PG_1-5_	A linear relationship between dose of MTX and PG_1–5_ was observed (ρ = n/a)
Brooks, 2007, *Therapeutic Drug Monitoring*^20^	18	NZ	24–80	72	37	78	15–25†; at least 12 weeks	Steroid (39)	Cross-sectional	HPLC –UV (day 0, 14 and 28) of MTX-PG_1-5_	Dose-dependent increase of MTX-PG_4_ and MTX-PG_(4,5)_ and MTX-PG _(3,4,5)_ (ρ = n/a) No relationship between MTX-PG_n_ and eGFR
Egan, 1999, *Alimentary Pharmacology and Therapeutics*^21^	30	USA	21–71	53	n/a	0	15 (60%) 25 (40%)	Steroid (100)	Prospective cohort of 16 weeks	Competitive protein binding assay of MTX-PG_total_	Significant increase of MTX-PG_total_ after dose escalation

[A] = abstract only. ADA = adalimumab. CD = Crohn’s disease. IFX = infliximab. mg/wk = milligram per week. MTX-PG = methotrexate polyglutamate. N = number of patients included. NZ = New Zealand. UK = United Kingdom. USA = United States of America. † = minimum – maximum. ǂ = mean ± standard deviation. § = information derived by contact author

Additionally, we compared our CD patients with RA patients using MTX for nine months, enrolled in the tREACH (n = 177) and MTX-R cohort (n = 59), where we measured MTX-PG concentrations previously with the same laboratory technique [15]. The median MTX-PG_total_ concentration and distribution between the polyglutamate subspecies (MTX-PG_1-5_) were comparable between the two diseases. This suggests that the results of pharmacokinetic RA studies could be translated to CD patients, which underscores the possibility of using MTX TDM across immune-mediated inflammatory diseases.

Surprisingly, our CD patients on subcutaneous MTX were found to have a higher interpatient variability of MTX-PG_total_ concentration than those on oral MTX. This is counterintuitive, as absorption of oral drugs is known to be more complex and variable than subcutaneously administered drugs [27].

This is the first study in CD patients that specifically focussed on pharmacokinetic parameters of MTX-PG accumulation. We demonstrate that the route of administration is an important determinant of the MTX-PG concentration, especially for MTX-PG_(4,5)_. Our findings are in line with results in RA patients. Dervieux et al*.* reported that the use of parenteral MTX in RA patients was associated with significantly higher MTX-PG_3_, MTX-PG_(4,5)_, and MTX-PG_total_ concentrations compared to oral administration of MTX [28]. Furthermore, Stamp et al*.* switched 30 RA patients from oral MTX to subcutaneous MTX and found significantly higher MTX-PG_(3–5)_ and MTX-PG_total_ concentrations after 24 weeks, particularly with regard to MTX-PG_(4,5)_.[29]

The lower long-chain MTX-PG concentration in oral users of MTX has been attributed to the observation that absorption from the gut is saturable at dosages above 15 mg, resulting in decreased bioavailability [30]. More recently, it has also been suggested that decreased oral bioavailability could result from a high load of microbial FPGS, which leads to polyglutamylation of MTX in the intestinal lumen, thereby hampering systemic uptake [31].

Since the route of administration can easily be changed, this determinant becomes clinically relevant. In an explorative survey in two Dutch University Medical Centres, 40–50% of CD patients on MTX maintenance therapy were found to use the oral formulation (data not published). Therefore, provided that the relationship of MTX-PG_(4,5)_ concentration and disease activity would be established, it might be worthwhile to switch non-responding CD patients with a low MTX-PG_(4,5)_ level from oral to subcutaneous MTX therapy. Conversely, in non-responders with high MTX-PG_(4,5)_ concentrations, a switch to a different class of drugs should be considered. The relation between the route of administration, the reached intracellular MTX-PG levels and MTX’s clinical efficacy should be further explored to support clinical decision making in this setting.

We confirm previous observations in patients with RA that a higher age is associated with increased concentrations of MTX-PG_(4,5)_ and MTX-PG_total_.[16, 28] A possible explanation could be that the enzyme GGH loses activity with advancing age, resulting in less deconjugation of long-chain MTX-PGs. Morgaceva et al*.* suggested that, amongst other reasons, altered body compositions and less intracellular water could explain the age-dependent distribution of the water-soluble MTX molecule [32]. Provided that the relationship of MTX-PG concentration and disease activity can be established, this implies that younger patients need a higher dose of MTX and preferably subcutaneous administration to reach therapeutic MTX-PG levels.

Renal function was found to be associated with the MTX-PG_total_ concentration as well. Although the study of Brooks et al*.* in IBD patients did not find a relationship between eGFR and the MTX-PG concentration [20], several RA studies with larger sample sizes observed an inverse relationship [28, 33]. As plasma MTX is mainly eliminated by the proximal tubules, lower eGFR could result in increased residence time of plasma MTX with the opportunity of a greater cellular uptake for polyglutamylation [32]. As all our patients had an eGFR > 60 ml/min/1.73 m^2^, a clinically useful cut-off point could not be determined. Nevertheless, if a pharmacokinetic model for MTX in CD patients would be developed, the eGFR of a patient should be included as a variable.

Baseline intracellular folate is thought to be an important determinant, as it reflects the capacity of transporters and MTX metabolizing enzymes to pump and keep both folates and MTX-PGs in the cell [16]. The positive correlation between the erythrocyte folate concentration and MTX-PG_5_ concentration (Fig. **2****c**) in our study is in line with this concept. However, other factors might affect the intracellular folate and MTX-PG concentrations during therapy as well, due to folic acid supplementation.

In our cohort, studying the dose of MTX as a determinant was hampered because 16 out of 19 patients used the same dose. However, it could still be an important determinant, as supported by our observed positive trend (Additional file 3: Figure), and by previous studies in patients with IBD and RA which show a clear positive correlation between MTX dose and MTX-PG concentration [17, 19–21, 33]. Furthermore, we found a trend toward lower MTX-PG_total_ and MTX-PG_n_ concentrations in smokers. In RA, it has been shown that smoking is associated with a significantly lower accumulation of erythrocyte MTX-PG [33]. Since MTX dosing and smoking behaviour might both affect MTX-PG concentrations, modulation of these factors might result in a higher efficacy of the drug and deserves future study.

Our findings should be interpreted with caution due to the small sample size, as determinants with a low prevalence could lead to type II errors. Secondly, the small sample size prevented us from analysing genetic determinants, such as polymorphisms in the FPGS enzyme and ABC-transporters. Another limitation is the cross-sectional study design, which amongst others, precludes elucidation of the role of baseline erythrocyte folate concentrations and the pharmacodynamic characteristics of MTX-PG_._ In our population, MTX-PG_n_ and MTX-PG_total_ concentration were not associated with either disease activity at baseline (*p* > 0.05) or disease activity at one year (data not shown). All previous studies measuring MTX-PG concentrations in patients with IBD did draw conclusions on the association of MTX-PG with disease activity, despite their cross-sectional study design [17, 18, 20]. Of note, the majority of studies included both CD and ulcerative colitis (UC) patients, and patients on concomitant TNF-α inhibitors, which impedes meaningful analysis of drug efficacy [17, 19–21]. Third, a cross-sectional design prevents the analysis of MTX-PG accumulation over time. Erythrocyte MTX-PG_(2,3)_ concentration reaches steady-state after approximately 2–3 months and erythrocyte MTX-PG_(4,5)_ concentration after 4–6 months [15]. As all our patients used MTX for a minimum of 7 months, we could not draw meaningful conclusions on the relationship between MTX-PG concentration and the duration of MTX use. At last, we did not assesse adherence. However, the MTX-PG concentrations can be used to detect non-adherence as an undetectable concentration (one patient in our cohort) represents no drug use at all during several weeks. All other patients in our cohort had a MTX-PG concentration of at least 20 nmol/L which suggests adherence [34].

Despite these limitations, this is, to our knowledge, the largest study in adult CD patients measuring MTX-PG concentrations so far, as well as the first study in CD to thoroughly examine determinants of MTX-PG concentrations.

## Conclusions

Our findings show that parenteral MTX administration enhances polyglutamylation of MTX to MTX-PGs, which could be clinically relevant as insufficient responders to oral MTX with a low MTX-PG concentration, especially younger patients with a normal renal function, could rationally be switched to subcutaneous MTX to achieve better disease control, provided that the relationship of MTX-PG concentrations and disease activity would be established. As higher MTX-PGs may improve the anti-inflammatory effects of MTX, this prescription advice could prevent a switch to costly biologicals.

We provide pharmacokinetic information that may allow tailoring of MTX to individual patients' characteristics, depending on target levels of MTX-PG, which are to be established in an ongoing prospective longitudinal study. Such pharmacokinetic and pharmacodynamic information may enable TDM of MTX in patients with CD aiming to optimise and individualise treatment with a more rational and extended use of MTX.

## Supplementary Information

**Additional file 1. Table:** Determinants of erythrocyte MTX-PG_n_ and MTX-PG_total_ concentrations.

**Additional file 2. Figure:** Concentration of erythrocyte MTX-PG_n_ for smokers (green) and non-smokers (orange).

**Additional file 3. Figure:** Boxplots of erythrocyte MTX-PG_total_ concentration for patients using different MTX doses.

## Data Availability

The datasets used and/or analysed during the current study are available from the corresponding author on reasonable request.

## Abbreviations

ABC: ATP-binding cassette
BMI: Body mass index
CV: Coefficient of variation
CD: Crohn’s disease
eGFR: Estimated glomerular filtration rate
FPGS: Folylpolyglutamate synthetase
GGH: ɣ-Glutamyl hydrolase
IBD: Inflammatory bowel disease
JIA: Juvenile idiopathic arthritis
MTX: Methotrexate
MTX-PG: Methotrexate polyglutamate
RA: Rheumatoid arthritis
TDM: Therapeutic drug monitoring
TNF: Tumour necrosis factor (TNF)
