# Supplementary material for: Subcutaneous administration, higher age and lower renal function are associated with erythrocyte methotrexate accumulation in Crohn’s disease: a cross-sectional study
Source: BMC Gastroenterol. 2022 Jul 30;22:365. doi: 10.1186/s12876-022-02439-y (PMC9338675; doi:10.1186/s12876-022-02439-y)
Supplement: Supplementary file 1 — Additional file 1. Table: Determinants of erythrocyte MTX-PGn and MTX-PGtotal concentrations. [file 12876_2022_2439_MOESM1_ESM.docx]

**Additional File 1**

**Table:**  **Determinants of erythrocyte MTX-PG_n_ and MTX-PG_total_ concentrations.**

| Determinants | MTX-PG_1_ | | | MTX-PG_2_ | | | | MTX-PG_3_ | | | | MTX-PG_4_ | | | | MTX-PG_5_ | | | | MTX-PG_total_ | | | |
| --- | --- | --- | --- | --- | --- | --- | --- | --- | --- | --- | --- | --- | --- | --- | --- | --- | --- | --- | --- | --- | --- | --- | --- |
|  | ρ | β | R^2^ | ρ | β | R^2^ | ρ | | β | R^2^ | ρ | | β | R^2^ | ρ | | β | R^2^ | ρ | | β | R^2^ |  |
| Age | 0.25 | 0.35 | - | 0.22 | 0.27 | - | **0.77 ^***^** | | **0.69**  **^**^** | **0.48**  **^**^** | **0.75**  **^***^** | | **0.78**  **^***^** | **0.61**  **^***^** | **0.71 ^**^** | | **0.76**  **^**^** | **0.58**  **^**^** | **0.80**  **^***^** | | **0.71 ^***^** | **0.50 ^***^** |  |
| Sex | **0.47^*^** | **0.47^*^** | - | 0.19 | 0.19 | - | 0.30 | | 0.30 | - | 0.25 | | 0.25 | - | 0.24 | | 0.24 | - | 0.43 | | 0.43 | - |  |
| Smoking status | -0.27 | -0.27 | - | **-0.54**  **^*^** | **-0.54 ^*^** | **0.29^*^** | -0.37 | | -0.37 | - | -0.31 | | -0.31 | - | -0.10 | | -0.10 | - | -0.37 | | -0.37 | - |  |
| BMI | 0.31 | -0.02 | - | 0.23 | 0.34 | - | -0.14 | | -0.18 | - | -0.23 | | 0.00 | - | -0.19 | | -0.04 | - | -0.10 | | -0.14 | - |  |
| eGFR | -0.32 | -0.35 | - | -0.18 | -0.14 | - | -0.47 | | -0.44 | - | **-0.68**  **^**^** | | **-0.61**  **^*^** | **0.37^*^** | -0.38 | | -0.40 | - | **-0.60**  **^**^** | | **-0.52**  **^*^** | **0.27^*^** |  |
| Creatinin | 0.17 | 0.42 | - | 0.05 | 0.09 | - | 0.09 | | 0.22 | - | 0.28 | | 0.27 | - | 0.14 | | 0.14 | - | 0.17 | | 0.36 | - |  |
| Erythrocyte folate | 0.33 | 0.18 | - | -0.16 | -0.08 | - | 0.02 | | 0.02 | - | 0.08 | | 0.11 | - | **0.66^*^** | | **0.68^*^** | **0.47^*^** | 0.20 | | 0.13 | - |  |
| Use of TNF-α inhibitor | 0.18 | 0.18 | - | -0.40 | -0.40 | - | -0.26 | | -0.26 | - | -0.23 | | -0.23 | - | -0.03 | | -0.03 | - | -0.10 | | -0.10 | - |  |
| MTX dose | 0.41 | 0.08 | - | 0.54 | 0.45 | - | 0.27 | | 0.21 | - | 0.22 | | 0.12 | - | -0.31 | | -0.23 | - | 0.30 | | 0.19 | - |  |
| Folic acid dose | -0.18 | -0.30 | - | -0.15 | -0.14 | - | -0.00 | | 0.02 | - | -0.01 | | 0.11 | - | 0.20 | | 0.15 | - | -0.03 | | -0.13 | - |  |
| Route of administration | 0.14 | 0.14 | - | -0.18 | -0.19 | - | **0.51^*^** | | **0.51^*^** | - | **0.69**  **^**^** | | **0.69 ^**^** | **0.48 ^**^** | **0.69**  **^**^** | | **0.69**  **^**^** | **0.47**  **^**^** | **0.47^*^** | | **0.47^*^** | **-** |  |
| Duration of MTX use | -0.01 | 0.34 | - | -0.12 | -0.19 | - | -0.11 | | -0.01 | - | -0.03 | | -0.04 | - | -0.08 | | -0.03 | - | -0.11 | | 0.15 | - |  |

Significant values are bold. * p < 0.05, ** p ≤ 0.01, *** p ≤ 0.001

Abbreviations: eGFR: estimated glomerular filtration rate. MTX: methotrexate. MTX-PG: methotrexate polyglutamate. TNF: Tumor necrosis factor.

Rho (ρ) represents the Point-Biserial correlation coefficient for dichotomous variables (sex, smoking status, disease activity, use of TNF-α inhibitor, route of administration MTX) whereas it represents the Spearman’s rank correlation coefficient for continuous or ordinal variables. Beta (β) is the standardized beta derived from univariable linear regression analysis. The R squared (R^2^) represents the explained variability derived from univariable linear regression analysis. R^2^ only shown if variable was significant at univariable analysis and at the comparison between two groups (last for dichotomous variables only).
