# Supplementary material for: Subcutaneous administration, higher age and lower renal function are associated with erythrocyte methotrexate accumulation in Crohn’s disease: a cross-sectional study
Source: BMC Gastroenterol. 2022 Jul 30;22:365. doi: 10.1186/s12876-022-02439-y (PMC9338675; doi:10.1186/s12876-022-02439-y)
Supplement: Supplementary file 2 — Additional file 2. Figure: Concentration of erythrocyte MTX-PGn for smokers (green) and non-smokers (orange). [file 12876_2022_2439_MOESM2_ESM.docx]

**Additional File 2**

**Figure: Concentration of erythrocyte MTX-PG_n­_ for smokers (green) and non-smokers (orange).**


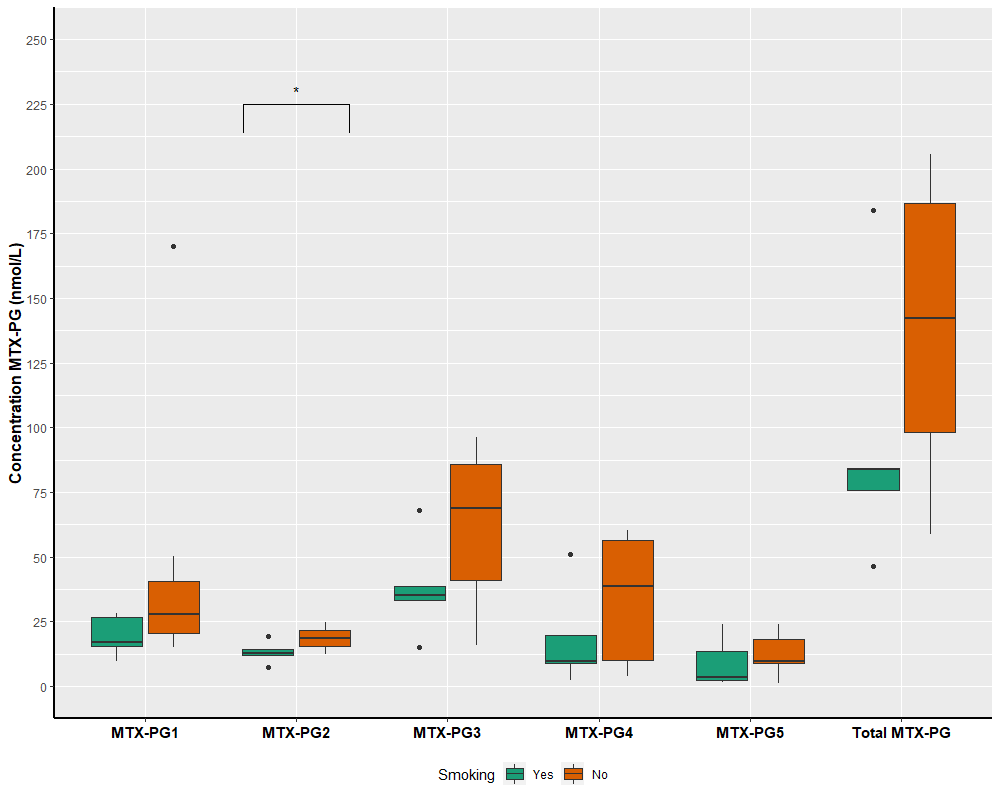


Significant difference tested by Mann Whitney *U* test (p = 0.08, p = 0.03, p = 0.09, p = 0.28, p = 0.73, p = 0.09, from left to right). Dots represent outliers.
