# Supplementary material for: Subcutaneous administration, higher age and lower renal function are associated with erythrocyte methotrexate accumulation in Crohn’s disease: a cross-sectional study
Source: BMC Gastroenterol. 2022 Jul 30;22:365. doi: 10.1186/s12876-022-02439-y (PMC9338675; doi:10.1186/s12876-022-02439-y)
Supplement: Supplementary file 3 — Additional file 3. Figure: Boxplots of erythrocyte MTX-PGtotal concentration for patients using different MTX doses. [file 12876_2022_2439_MOESM3_ESM.docx]

**Additional File 3**

**Figure: Boxplots of erythrocyte MTX-PG_total_ concentration for patients using different MTX doses.**


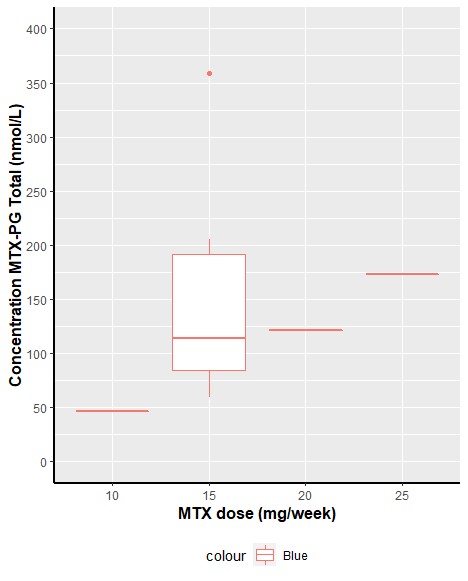


Number of patients using 10 (n=1), 15 (n=16), 20 (n=1) or 25 (n=1) mg/week.
Significant difference tested by Kruskal Wallis test (p of MTX-PG_total_ concentration = 0.42). Dot represents outlier.
